# Association of NAD^+^ levels with metabolic disease in a community-based study

**DOI:** 10.3389/fendo.2023.1164788

**Published:** 2023-04-20

**Authors:** Yuhe Liu, Xueyu Chen, Xuan Deng, Fan Yang, Jinping Zheng, Tianyun Zhou, Ling Xu, Xiaomei Xie, Zhenyu Ju, Baoguo Wang, Caiping Zhang, Yong Zhou

**Affiliations:** ^1^ Institute of Biochemistry and Molecular Biology, Hengyang Medical College, University of South China, Hengyang, Hunan, China; ^2^ Department of Biostatistics, School of Public Health, Cheeloo College of Medicine, Shandong University, Jinan, China; ^3^ Clinical Research Institute, Shanghai General Hospital, Shanghai Jiao Tong University School of Medicine, Shanghai, China; ^4^ The First Affiliated Hospital of Jinan University, Institute of Aging and Regenerative Medicine, Jinan University, Guangzhou, China; ^5^ Department of Public Health and Preventive Medicine, Changzhi Medical College, Changzhi, China; ^6^ Clinical Medicine, School of Basic Medicine, Shanghai Medical College Fudan University, Shanghai, China; ^7^ Department of Clinical Laboratory, Tangshan Gem Flower Hospital, Tangshan, China; ^8^ Sanbo Brain Hospital, Capital Medical University, Beijing, China

**Keywords:** nicotinamide adenine dinucleotide, metabolic disease, cross-sectional study, whole blood, population

## Abstract

**Background:**

Nicotinamide adenine dinucleotide (NAD^+^) is a coenzyme and plays a crucial role in several metabolic processes. This study explored the association of nicotinamide adenine dinucleotide (NAD^+^) levels with metabolic disease (MD) in adults.

**Methods:**

In this cross-sectional study, all data were collected from the Jidong community. MD was defined as the presence of one or more of the following disease components: hypertension, dyslipidemia, diabetes, hyperuricemia, obesity, and non-alcoholic fatty liver disease (NAFLD). The MD components were categorized into three groups: those with one component, those with two components, and those with three to six components. The whole blood NAD^+^ level was measured using a cycling assay and LC-MS/MS analysis. The participants were divided into four groups based on their NAD^+^ level quartiles. Multivariable logistic regression was used to evaluate the association of the whole blood NAD^+^ levels with MD.

**Results:**

Of the 1,394 eligible participants, the average age was 43.2 years, and 74.3% had MD. In the top quartile of NAD^+^, the prevalence of MD and each of its components (hypertension, hyperlipidemia, diabetes, hyperuricemia, obesity, and NAFLD) were 87.9% 35.2%, 62.3%, 8.7%, 36.9%, 21.0%, and 60.5%, respectively. As compared with the lowest NAD^+^ quartile (≤29.4 μmol/L), the adjusted odds ratios and 95% confidence interval of the highest quartile were 3.01 (1.87-4.87) for MD, 2.48 (1.44-4.29) for 1 MD component, 2.74 (1.45-5.17) for 2 MD components, and 4.30 (2.32-7.98) for 3-6 MD components. The risk of MD began to increase at NAD^+^ levels of 31.0 μmol/L, as revealed by the gradient associations of NAD^+^ levels with MD. There was no significant interaction between age, sex, drinking, smoking, and NAD^+^ for MD (p for interaction ≥0.10).

**Conclusions:**

Increased NAD^+^ was significantly associated with MD, as well as its individual components. Our findings provide new evidence for the relationship between blood NAD^+^ levels and MD.

## Introduction

Metabolic disease (MD) has become a significant global health concern due to its increasing incidence and burden on human health (1). The disease typically results from abnormalities in the metabolism of substances or energy, such as diabetes, hypertension, and non-alcoholic fatty liver disease (NAFLD) (2, 3). In 2015, metabolic abnormalities in blood glucose, blood pressure, and lipids contributed to over 24 million deaths worldwide (4). Hyperuricemia, which affects more than 20% of US adults according to data from 2007-2008, is also becoming increasingly prevalent and is considered the second most common MD in China after diabetes (5, 6). Chronic MD can have multiple adverse consequences. NAD^+^, a pyridine nucleotide, was first discovered by regulating the metabolic rate of yeast extracts, and later was well-known for its role in redox reactions. It has emerged as a key regulator of a variety of metabolic processes in cells, encompassing glycolysis, the TCA cycle, oxidative phosphorylation, DNA repair, and gene expression. Accordingly, this highlights the critical role of NAD^+^ in maintaining proper metabolic function (7, 8) and the critical role NAD^+^ plays in maintaining proper metabolic function.

Changes in NAD^+^ levels have been associated with metabolic dysfunction (9). Several studies have reported that NAD^+^ plays an important role in regulating processes associated with the pathogenesis of obesity, NAFLD (10), diabetes (11, 12), and hypertension (2, 13). Enhanced NAD^+^ levels may alleviate symptoms of NAFLD (14). However, several studies have proposed that elevated levels of NAD^+^ precursors have been significantly associated with diabetes (15) and cardiovascular disease (16, 17). Additionally, in an animal study, nicotinamide mononucleotide (NMN), a NAD^+^ precursor, impaired the benefits of exercise on glucose metabolism in diet-induced obesity (18). Therefore, the association of NAD^+^ with clinical diseases, particularly MD, remains controversial. Up to now, no study has evaluated the association of NAD^+^ with multiple common chronic diseases. As such, the current study aimed to investigate the association between NAD^+^ levels and MD, including hypertension, hyperlipidemia, diabetes, hyperuricemia, obesity, and NAFLD, in a large, community-based cross-sectional study.

## Methods

### Study design and participants

**Figure 1:**
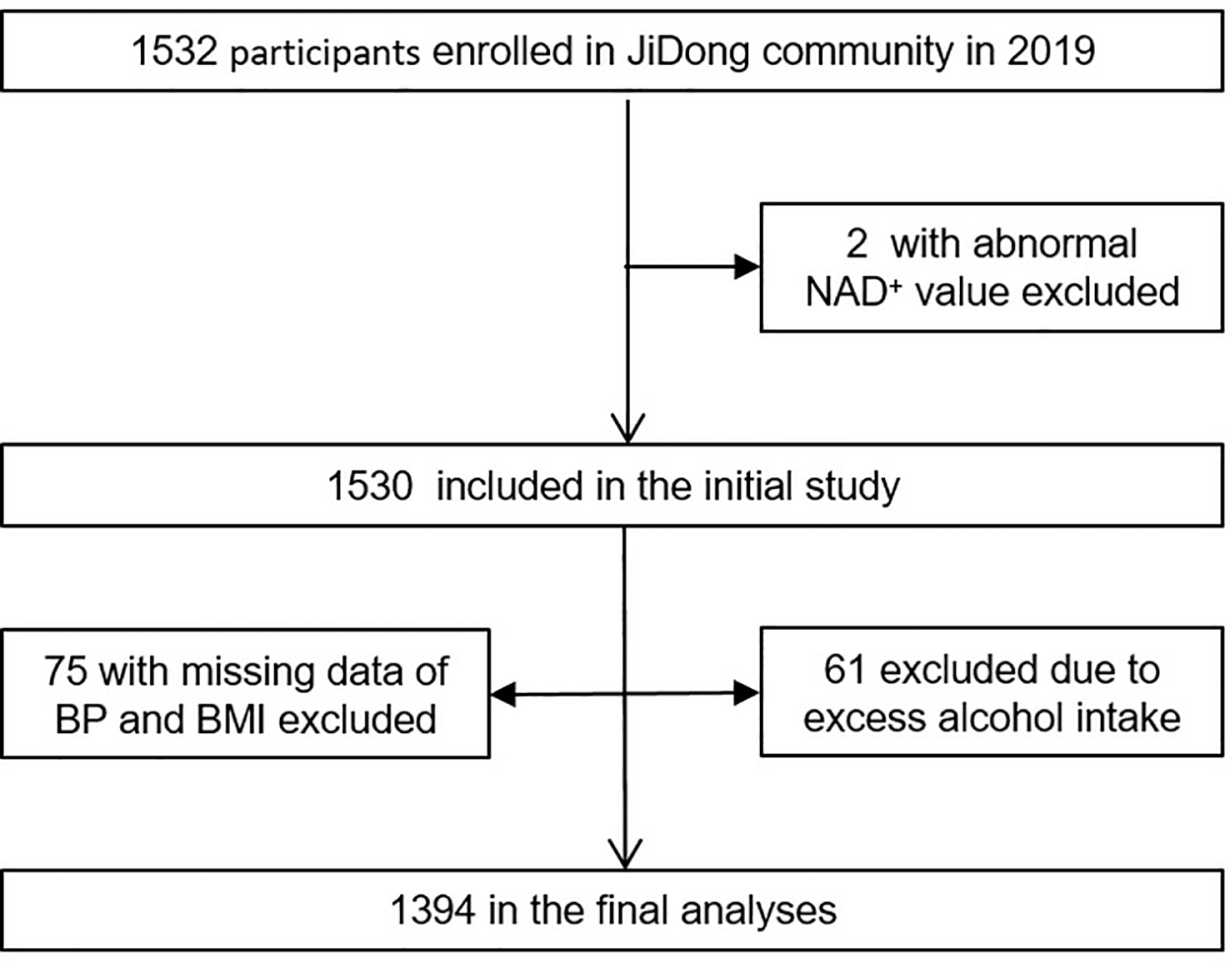
Flow chart of this study. BP, blood pressure; BMI, body mass index.

The participants were recruited from the Jidong community in Tangshan, a large modern city in northern China. From 2019 to 2020, a total of 1,532 participants were recruited for the study after excluding participants with an abnormal value of NAD^+^ (n=2), missing data on blood pressure (BP) and body mass index (BMI) (n=75), or having excess alcohol intake (n=61) (**Figure 1**). All participants provided informed consent, and the study was conducted in accordance with the Helsinki Declaration and approved by the Ethical Committees of the Staff Hospital of Jidong Oilfield of China National Petroleum Corporation.

### Data collection

Participants were interviewed and completed a structured questionnaire on demographic characteristics, smoking habits, alcohol consumption, and medical history. Exercise frequency was classified as “Inactive”, “Moderately active”, and “Very active”. Education level was categorized as “Middle school or below” or “college or above”. BMI was calculated by dividing measured weight in kilograms by the square of measured height in meters and was categorized as “<18.5”, “18.5-23.9”, “24.0-27.9”, and “≥28.0”. Blood pressure was measured using an automatic digital blood pressure monitor. The intake of meat and vegetables in the daily diet was categorized as “Never”, “Occasionally”, and “Very often”.

### Measurement of NAD^+^ levels

After overnight fasting, blood samples were collected from the large antecubital veins, preserved in EDTA tubes (ethylene diamine tetraacetic acid), and NAD^+^ levels were determined in the laboratory by cycling assay and LC-MS/MS analysis. The cycling assay mainly applies the biochemical basis of NAD^+^, which transitions back and forth between redox states during the assay. LC-MS/MS analysis is a powerful tool for detecting compounds’ qualitative and quantitative analysis. These methods are consistent with our previous study (19). Finally, the data measured by the cycling assay and LC–MS/MS are compared and validated to ensure the reliability of the data.

### Assessment of metabolic disease

MD was defined as the presence of one or more of the following disease components: hypertension, dyslipidemia, diabetes, hyperuricemia, obesity, and NAFLD. Hypertension was defined as systolic blood pressure (SBP) ≥140mmHg or diastolic blood pressure (DBP) ≥90mmHg or self-reported taking antihypertensive medication or having been diagnosed with hypertension (20). Dyslipidemia was defined as serum total cholesterol ≥5.7 mmol/L, triglyceride≥1.7 mmol/L, low-density lipoprotein cholesterol level ≥4.1 mmol/L any use of lipid-lowering drugs, or any self-reported history of dyslipidemia. Diabetes was defined as fasting glucose ≥7.0 mmol/L, any use of glucose-lowering drugs, or any self-reported history of diabetes (21). Hyperuricemia was defined as a serum uric acid (SUA) level above 420 μmol/L in men and above 357 μmol/L in women (22). Obesity was defined as a body mass index ≥28 kg/m^2^ (23). NAFLD was defined as diffusely increased echogenicity of the liver relative to the kidney, ultrasound beam attenuation, and poor visualization of intrahepatic structures, excluding those with excess alcohol intake and other liver diseases (24).

### Statistical analysis

Participants were divided into four groups by the quartile of NAD^+^ levels. Normally distributed continuous variables were expressed as mean ± standard deviation (SD) and analyzed using one-way analysis of variance (ANOVA). Categorical variables are shown as frequencies and percentages and analyzed using the chi-square test. Logistic regression analysis was used to analyze the relationship between NAD^+^ levels and MD.

Additionally, the number of MD components was categorized as “1 component”, “2 components”, and “3-6 components”. Multivariate generalized linear regression analysis and restricted cubic splines (RCS) were used to determine the association of NAD^+^ levels with MD components after adjusting for age, gender, and the number of red blood cell counts (RBC), smoke, drink, exercise, education, and the meat diet. We performed the same association analysis in a population stratified by age, sex, drink, and smoke, grouping by NAD^+^ cutoff value and NAD^+^ quartiles, respectively. All statistical analyses were two-sided and the level of significance was α=0.05. The statistical analyses were performed using SAS software, version 9.4 (SAS Institute Inc., Cary, NC, USA).

## Results

### Baseline characteristics of eligible participants

Out of the 1,532 participants with NAD^+^ data, 1,394 participants were ultimately analyzed after meeting our inclusion and exclusion criteria (**Figure 1**). **Table 1** displays the demographic characteristics of all included participants, stratified by quartiles of NAD^+^ levels. The mean age of participants was 43.2 years, with 52.7% being male. The proportion of participants with MD was 74.3%, with a gradual increase observed from quartile 1 to quartile 4 of NAD^+^ levels. Significant differences were found among NAD^+^ quartile groups in terms of gender, smoking habits, drinking habits, BMI, SBP, DBP, TG, LDL-C, and HDL-C (all p values <0.001). Mean RBCs were higher in quartile 3 and quartile 4 compared to quartile 1 and quartile 2, while there were no significant differences in mean total cholesterol levels, exercise frequency, meat diet, and vegetable diet.

**Table 1:** Baseline characteristics of eligible participants according to NAD^+^ levels in the study.

Characteristics	Overall (n = 1394)	NAD^+^ levels				P-value
		Quartile 1	Quartile 2	Quartile 3	Quartile 4	
		< 29.4 (n = 348)	29.4 - 32.8 (n = 353)	32.8 - 36.4 (n = 346)	≥ 36.4 (n = 347)	
Age, mean ± SD, y	43.2 ± 11.3	43.8 ± 11.0	44.1 ± 12.0	42.6 ± 11.3	42.4 ± 10.9	0.12
Male, No (%)	735 (52.7)	107 (30.8)	168 (47.6)	211 (61.0)	249 (71.8)	< 0.001
Exercise frequency, No (%)						0.50
Inactive	361 (27.1)	98 (29.3)	78 (23.2)	93 (28.1)	92 (27.7)	
Moderately active	245 (18.4)	62 (18.6)	60 (17.8)	57 (17.2)	66 (19.9)	
Very active	728 (54.6)	174 (52.1)	199 (59.1)	181 (54.7)	174 (52.4)	
Education level, No (%)						0.007
Middle school or below	381 (27.3)	114 (32.8)	105 (29.8)	85 (24.6)	77 (22.2)	
College or above	1013 (72.7)	234 (67.2)	248 (70.3)	261 (75.4)	270 (77.8)	
Current smoking, No (%)	324 (23.2)	42 (12.1)	72 (20.4)	85 (24.6)	125 (36.0)	< 0.001
Current drinking, No (%)	254 (18.2)	37 (10.6)	58 (16.4)	72 (20.8)	87 (25.1)	< 0.001
Metabolic disease, No (%)	1036 (74.3)	220 (63.2)	251 (71.1)	260 (75.1)	305 (87.9)	< 0.001
BMI, kg/m^2^						< 0.001
< 18.5	38 (2.7)	12 (3.5)	13 (3.7)	8 (2.3)	5 (1.4)	
18.5-23.9	592 (42.5)	185 (53.2)	167 (47.3)	134 (38.7)	106 (30.6)	
24.0-27.9	552 (39.6)	124 (35.6)	123 (34.8)	142 (41.0)	163 (47.0)	
≥ 28.0	212 (15.2)	27 (7.8)	50 (14.2)	62 (17.9)	73 (21.0)	
SBP, mean ± SD, mmHg	125.1 ± 17.1	122.8 ± 17.4	123.7 ± 17.9	126.2 ± 16.8	127.8 ± 15.8	< 0.001
DBP, mean ± SD, mmHg	80.3 ± 12.9	77.6 ± 13.1	78.7 ± 12.5	81.4 ± 12.2	83.6 ± 13.1	< 0.001
FBG, mean ± SD, mmol/L	5.6 ± 1.3	5.4 ± 1.0	5.6 ± 1.3	5.7 ± 1.5	5.7 ± 1.4	0.04
TG, mean ± SD, mmol/L	1.8 ± 1.5	1.5 ± 1.0	1.6 ± 1.2	1.8 ± 1.2	2.3 ± 2.1	< 0.001
TC, mean ± SD, mmol/L	5.1 ± 1.0	5.1 ± 0.9	5.1 ± 1.0	5.2 ± 1.0	5.2 ± 1.0	0.070
UA, mean ± SD, μmol/L	346.5 ± 93.1	317.8 ± 88.3	333.9 ± 82.9	353.9 ± 95.7	380.8 ± 93.4	< 0.001
LDL-C, mean ± SD, mmol/L	2.3 ± 0.7	2.2 ± 0.7	2.2 ± 0.7	2.4 ± 0.8	2.4 ± 0.8	< 0.001
HDL-C, mean ± SD, mmol/L	1.2 ± 0.3	1.3 ± 0.3	1.2 ± 0.3	1.2 ± 0.3	1.1 ± 0.2	< 0.001
RBC, mean ± SD, 10^12^/L	4.8 ± 0.5	4.6 ± 0.5	4.7 ± 0.5	4.8 ± 0.5	4.9 ± 0.5	< 0.001
Meat diet, No (%)						0.41
Never&Occasionally	968 (82.5)	232 (83.5)	235 (81.6)	236 (80.0)	265 (84.9)	
Very often	205 (17.5)	46 (16.6)	53 (18.4)	59 (20.0)	47 (15.1)	
Vegetable diet, No (%)						0.69
Never&Occasionally	76 (6.5)	22 (7.9)	16 (5.6)	19 (6.4)	19 (6.1)	
Very often	1096 (93.5)	255 (92.1)	272 (94.4)	276 (93.6)	293 (93.9)	

BMI denotes Body mass index; DBP, diastolic blood pressure; FBG, fasting blood glucose; HDL-C, high-density lipoprotein cholesterol; LDL-C, low-density lipoprotein cholesterol; NAD^+^, nicotinamide adenine dinucleotide; RBC, red blood cells; SBP, systolic blood pressure; TC, total cholesterol; TG, triglyceride; UA, uric acid.

### Prevalence of metabolic disease in the study population

**Figure 2:**
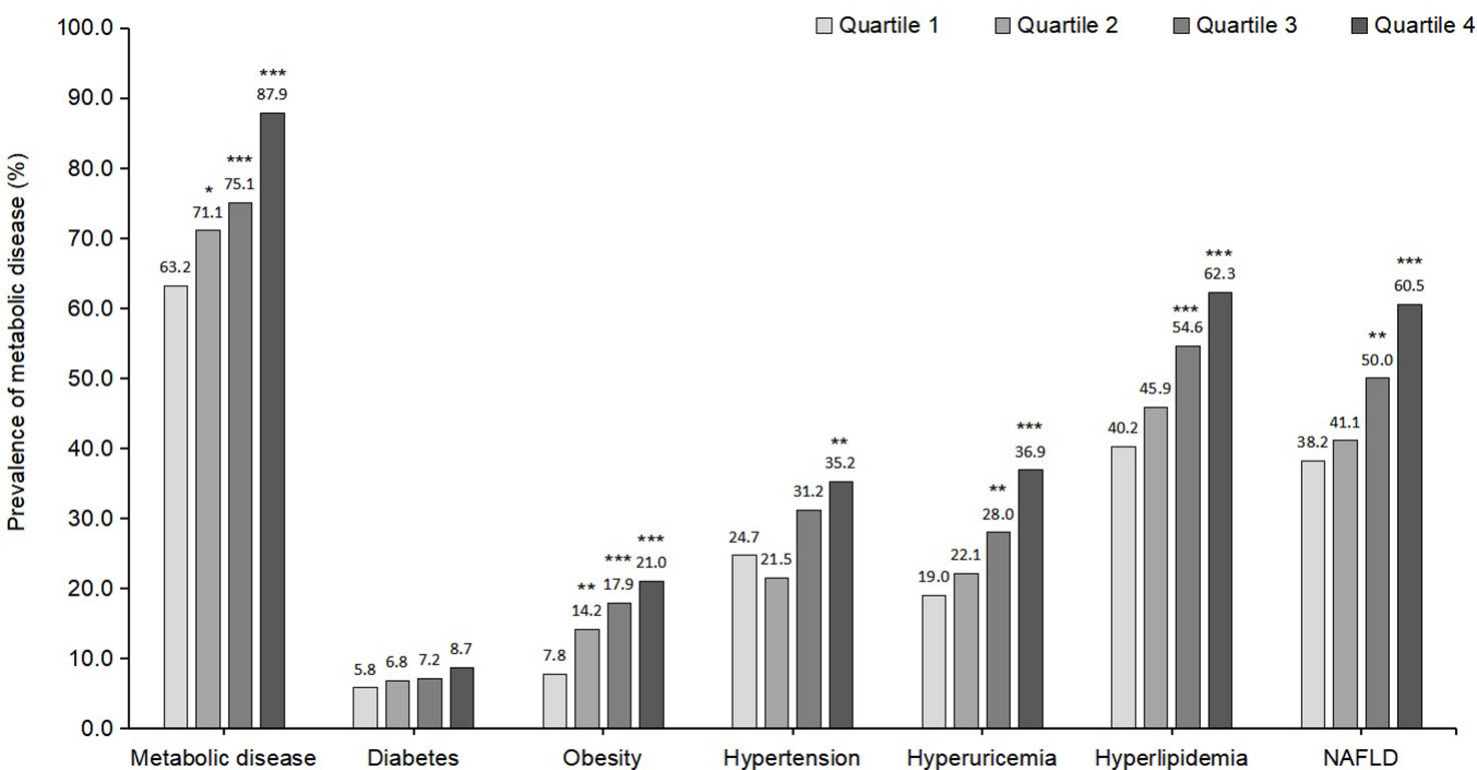
Prevalence of metabolic disease and each component of metabolic disease according to NAD^+^ levels. Metabolic disease is defined as hypertension, hyperlipidemia, diabetes, hyperuricemia, obesity, or NAFLD among the whole participants. NAFLD, non-alcoholic fatty liver disease; *indicates p < 0.05; **p < 0.01; ***p < 0.001; Comparison between 2^nd^, 3^rd^, 4^th^ *vs* 1^st^ quartile of the NAD^+^ levels.

**Figure 2** illustrates the prevalence of MD and its individual components across NAD^+^ categories. In the fourth quartile of NAD^+^, the overall percentage of MD was 87.9%. In the highest NAD^+^ quartile, the prevalence rates of MD, hypertension, hyperlipidemia, diabetes, hyperuricemia, obesity, and NAFLD were 87.9%, 35.2%, 62.3%, 8.7%, 36.9%, 21.0%, and 60.5%, respectively.

### Association of NAD^+^ levels with MD or its components

**Table 2** presents the association between NAD^+^ levels and MD in the entire population after adjusting for confounding variables. Compared to the first NAD^+^ quartile, the adjusted odds ratio for MD was 3.07 (1.91-4.95) in the highest quartile. **Table 3** displays the results of multinomial logistic regression analysis examining the association between NAD^+^ levels and MD components. Compared to the first NAD^+^ quartile, the adjusted odds ratio for the highest quartile was 2.49 (1.45-4.28) for the group with 1 MD component and 2.78 (1.48-5.24) for the group with 2 MD components. For the group with 3-6 MD components, the adjusted odds ratio for the highest NAD^+^ quartile was 4.44 (2.40-8.21), compared to the first quartile of NAD^+^ levels.

**Table 2:** Association of NAD^+^ levels with metabolic disease in the whole population.

NAD^+^ quartiles	Events, N (%)	Odds ratios (95% CI)		
		Unadjusted	Adjusted	
Quartile 1	220 (63.2)	Ref	Ref	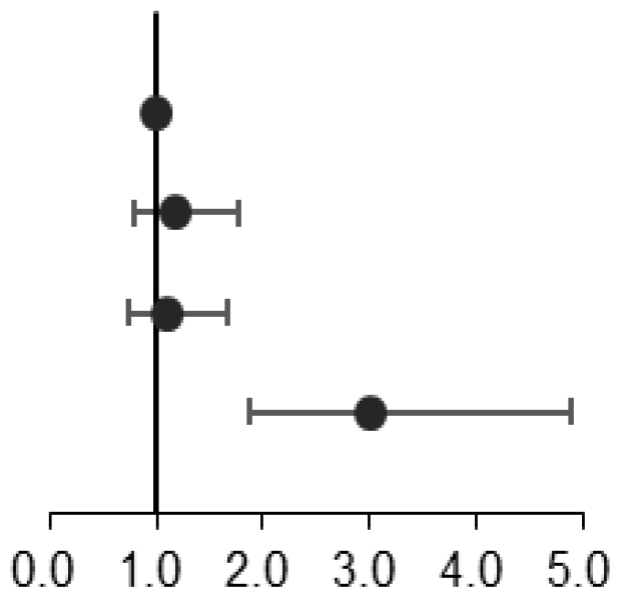
Quartile 2	251 (71.1)	1.43 (1.04-1.97)	1.20 (0.81-1.78)	
Quartile 3	260 (75.1)	1.76 (1.27-2.44)	1.12 (0.75-1.69)	
Quartile 4	305 (87.9)	4.23 (2.86-6.24)	3.07 (1.91-4.95)

Multivariable analysis adjusted for age, gender, the number of RBCs, smoke, drink, exercise, education, and the meat diet. OR, Odd Ratios; CI, confidence interval; Ref, reference.

**Table 3:** Association of NAD^+^ levels with metabolic disease components in the multivariable analysis among the whole population.

Metabolic disease components	Events, N (%)	Odds ratios (95% CI)		
		Unadjusted	Adjusted	
1 component vs 0 component				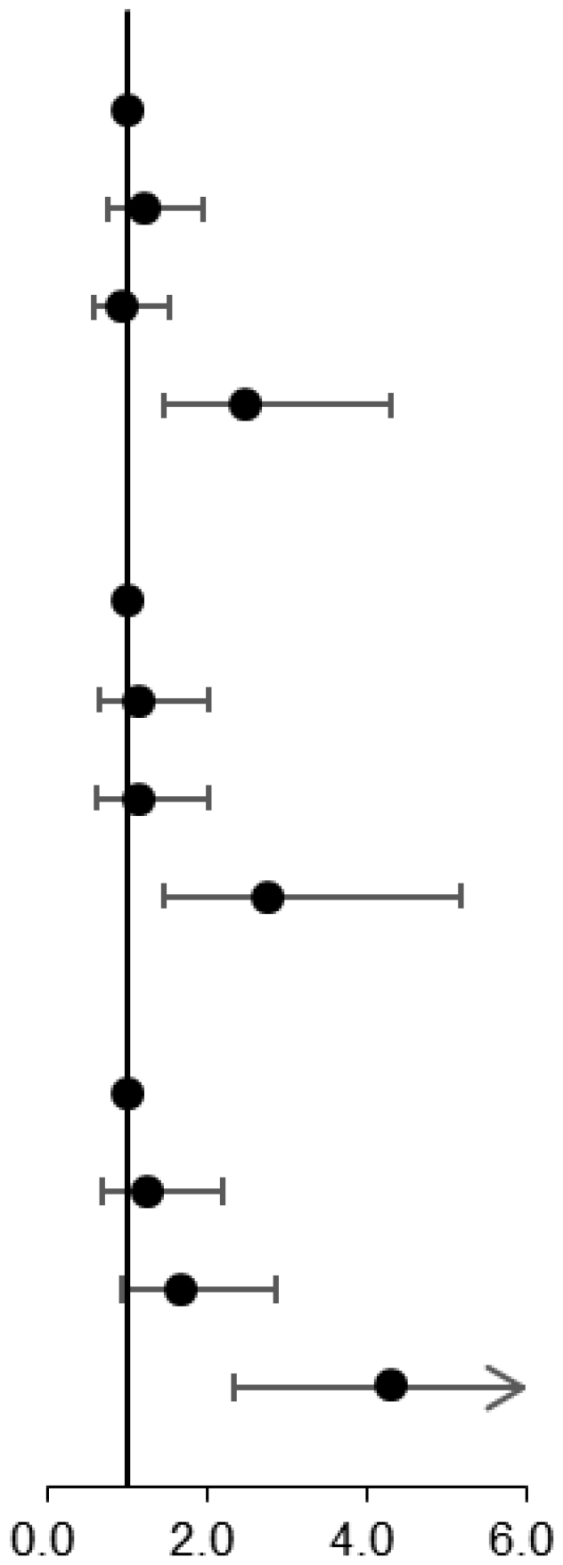
Quartile 1	87 (25.0)	Ref	Ref	
Quartile 2	97 (27.5)	1.40 (0.95-2.07)	1.19 (0.75-1.88)	
Quartile 3	71 (20.5)	1.22 (0.80-1.84)	0.95 (0.58-1.55)	
Quartile 4	79 (22.8)	2.77 (1.74-4.40)	2.49 (1.45-4.28)	
2 components vs 0 component				
Quartile 1	58 (16.7)	Ref	Ref	
Quartile 2	71 (20.1)	1.54 (0.99-2.37)	1.18 (0.68-2.07)	
Quartile 3	67 (19.4)	1.72 (1.10-2.68)	1.16 (0.65-2.07)	
Quartile 4	68 (19.6)	3.57 (2.18-5.86)	2.78 (1.48-5.24)	
3-6 components vs 0 component				
Quartile 1	75 (21.6)	Ref	Ref	
Quartile 2	83 (23.5)	1.39 (0.93-2.09)	1.27 (0.72-2.23)	
Quartile 3	122 (35.3)	2.42 (1.63-3.60)	1.67 (0.96-2.91)	
Quartile 4	158 (45.5)	6.42 (4.12-10.01)	4.44 (2.40-8.21)	

Multivariable analysis adjusted for age, gender, the number of RBCs, smoke, drink, exercise, education, and meat. CI, confidence interval, Ref, reference.

### Restricted cubic splines analysis for NAD^+^ in MD or its components

**Figure 3:**
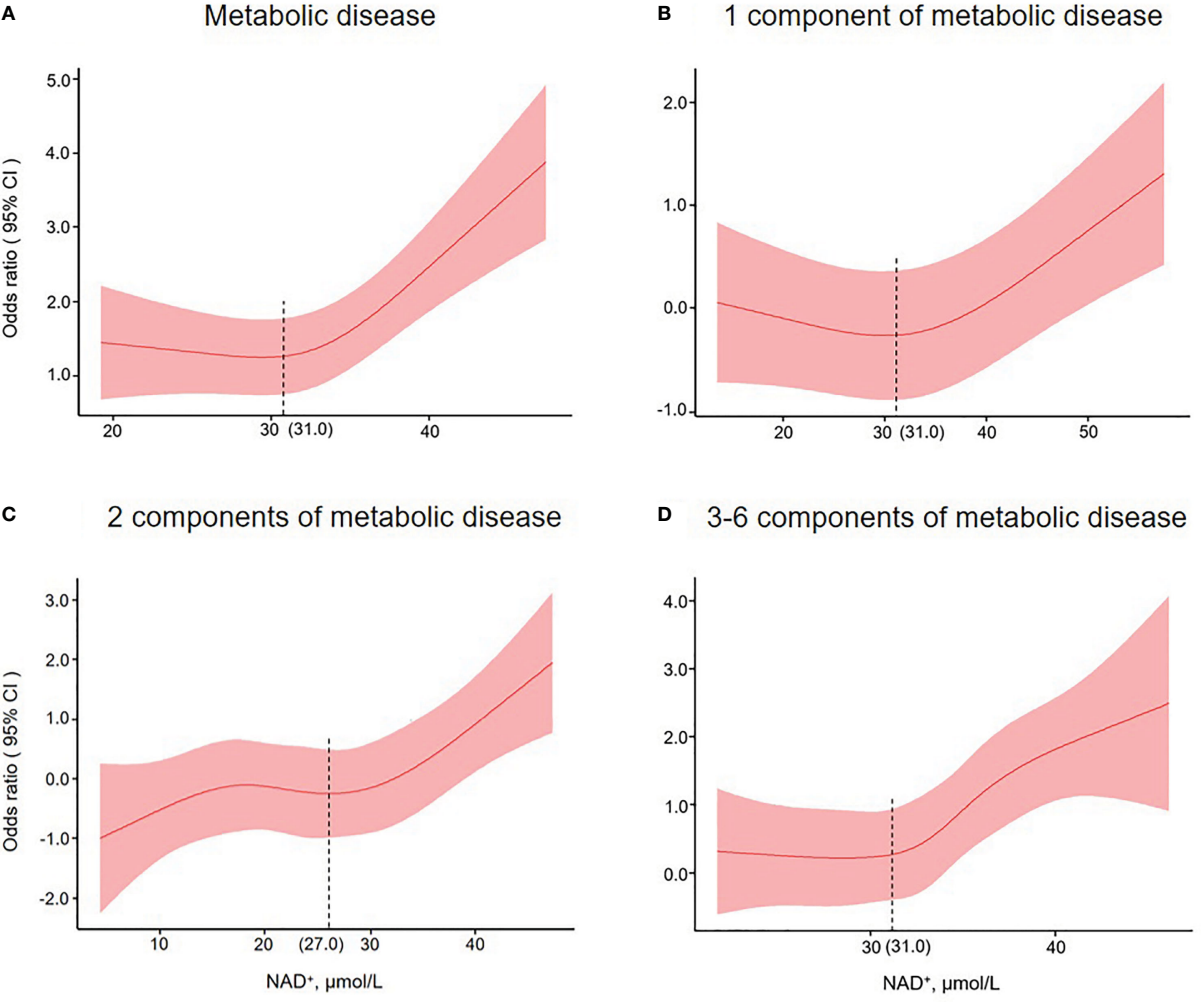
Restricted cubic spline plots for associations of NAD^+^ levels with metabolic disease **(A)**, 1 metabolic disease component **(B)**, 2 metabolic disease components **(C)**, and 3-6 metabolic disease components **(D)** in the population of interest. Models were adjusted for age, gender, the number of RBCs, smoking, drinking, exercise, education, and meat. The red line and shaded area represent the odds ratios and corresponding 95% confidence intervals.

To illustrate the relationship between NAD^+^ levels and MD, we utilized restricted cubic splines to depict the risk of MD or its components in a given population (**Figure 3**). Our findings indicate that the risk of MD remained constant in individuals with NAD^+^ levels below 31.0 μmol/L, but increased as the levels surpassed 31.0 μmol/L. For those with 1 MD component, the risk slightly decreased before the 31.0 μmol/L threshold but increased afterward. The risk of 2 MD components increased initially, plateaued before 27.0 μmol/L, and then continued to increase. Additionally, the risk of 3-6 MD components began to rise at the 31.0 μmol/L threshold.

### Stratified analysis for NAD^+^ in MD or its components

In order to assess the association of NAD^+^ level with MD, further subgroup analyses were performed by age, sex, drinking, and smoking. The results demonstrated that age, sex, drinking, and smoking did not significantly alter the associations between NAD^+^ levels and MD or its components (**Supplementary Table 1**). In addition, the results of the subgroup analysis based on the NAD^+^ cut-off values were consistent with the results of the NAD^+^ quartiles (**Supplementary Table 2**).

## Discussion

In the cross-sectional analysis, approximately 75% of the participants were found to have MD. Participants with elevated NAD^+^ levels of more than 36.4 μmol/L were observed to have a 3-fold higher risk of MD and about a 4.5-fold higher risk of 3-6 MD components, as compared to those with low NAD^+^ levels of less than approximately 31.0 μmol/L. Furthermore, the risk of MD and its components exhibited a rapid increase around the 31.0 μmol/L mark of NAD^+^ levels. These associations remained significant and were not significantly altered by age, sex, drinking status, or smoking status. Our findings provide compelling new evidence for the relationship between blood NAD^+^ levels and MD.

Our study revealed that the prevalence of MD was high, with more than 70% of the study population affected. In 2015, the prevalence of metabolic disease multimorbidity in China was about 30% (25). The reason for the higher prevalence in our was that our definition of MD, which includes at least one chronic disease, differs from the definition of metabolic disease multimorbidity, which requires the presence of two or more chronic conditions. A meta-analysis in 2016 reported the prevalence of metabolic syndrome to be approximately 25% in mainland China (26), while previous studies in other countries, such as the United States (35%) (27), Turkey (44%) (28), and Iran (37%) (29), have reported varying prevalence rates. These differences may be due to regional disparities, data collection methods, or sample population characteristics. In addition, we found that the percentage of diabetes and hypertension was 8.7% and 35.2% in the highest NAD^+^ quartiles, respectively. However, a previous prospective study reported percentages of diabetes (24.4%) and hypertension (87.9%) among individuals in the highest quartiles of NAD^+^ precursor level (30). The observed differences may be attributed to variations in the ethnicities of the populations studied. Therefore, further research is warranted to explore the prevalence of MD and its components in large cohort studies.

In our study, higher NAD^+^ levels were associated with an increased risk of MD and its components. In addition, we observed that there was a significant association of NAD^+^ levels with the number of metabolic disease components. A study found that pro-neurotensin/neuromedin N (pro-NT/NMN), another NAD^+^ precursor, was positively associated with incident metabolic syndrome (MetS) (31), which is consistent with our findings of the effect of NAD^+^ on hyperuricemia and NAFLD. Reasons for Geographic and Racial Differences in Stroke (REGARDS) cohort also showed that elevated systemic pro-NT/NMN was significantly associated with the risk of incident ischemic stroke in the whole population (32). A high level of N1-methyl nicotinamide, the NAD^+^ precursor, was also strongly associated with both diseases, obesity, and diabetes which is similar to our results (33). Extracellular nicotinamide phosphoribosyltransferase (eNAMPT), the rate-limiting enzyme in the NAD^+^ synthesis pathway, might play an important role in the pathogenesis of vascular inflammation in obesity and diabetes (34–36). In addition, we could not exclude the possibility that an increase in NAD would be compensatory under pathological conditions. Nevertheless, the results regarding the association of NAD^+^ with MD remain controversial and need to be further explored by the mechanism of biomedical sciences. It will be necessary for our findings to be verified by other large-scale, prospective, and longitudinal studies in the future.

The NAD^+^ dose-dependent analysis further supports the above-mentioned association of NAD^+^ levels with MD or its components. We found that the cut-off point of NAD^+^ was around 31.0 μmol/L, where the risk of MD began to increase more rapidly. In addition, it has to be mentioned that with increasing NAD^+^ levels, no statistical difference for MD components was observed, probably due to the limited sample size of our study. However, we did not find that the association of NAD^+ with^ MD was altered by sex, age, drinking, and smoking after analysis based on different subgroups of NAD^+^ levels. There may be differences due to sample size limitations, but the overall trend was consistent. A study has reported that NAD^+^ levels are markedly reduced when blood alcohol levels are high (37). Our previous research showed that NAD^+^ content declines with age, especially in males (19). In our subgroup analysis, the same conclusion was reached. Meanwhile, in this study, we found that relatively high levels of NAD^+^ were positively associated with MD compared to relatively low levels of NAD^+^.

The study had several limitations that need to be acknowledged. Firstly, as our analysis was based on cross-sectional data, we were unable to determine causality between MD and NAD^+^ levels. Secondly, while our study was the first of its kind to be conducted on a large scale in China, it was limited by the sample size. Thirdly, it is important to note that our findings may not be generalizable to other ethnicities or races since our study was conducted solely on participants from a northern Chinese city. Furthermore, we lack data on NAD^+^-related metabolic compounds in the present study, such as NADH, NAAD, NMN, NAMN, NAM, NA, ADPR, and 5’AMP. In future studies, we will explore the relationship between metabolic disease and compounds of NAD^+^ comprehensively and systematically.

This study indicated that there was a significant association between high blood NAD^+^ levels and MD and its components. Specifically, our findings show that the risk of MD starts to increase significantly when NAD^+^ levels reach 31.0 μmol/L. This novel evidence adds to understanding of the connections between blood NAD^+^ levels and MD.

## Data availability statement

The raw data supporting the conclusions of this article will be made available by the authors, without undue reservation.

## Ethics statement

The studies involving human participants were reviewed and approved by the Ethics Committee of the Jidong Oilfield Staff Hospital of China National Petroleum Corporation. The patients/participants provided their written informed consent to participate in this study.

## Author contributions

YZ and CZ conceived and designed the study and analyses. YL, XC, and XD analyzed data and drafted the paper. ZJ, FY and JZ washed the data. BW revised it critically for important intellectual content. TZ and XX performed the material preparation and data collection. XL carried out the critical revision of the article. YL, XC, and XD contributed equally. All authors contributed to the article and approved the submitted version.
