# Supplementary material for: Association of NAD+ levels with metabolic disease in a community-based study
Source: Front Endocrinol (Lausanne). 2023 Apr 20;14:1164788. doi: 10.3389/fendo.2023.1164788 (PMC10158491; doi:10.3389/fendo.2023.1164788)
Supplement: Supplementary file 1 [file Table_1.docx]

Supplementary table 1. Subgroups analysis for the association between NAD^+^ levels and metabolic disease or its components.

|  | Age | | |  | Sex | | |  | Drink | | |  | Smoke | | |
| --- | --- | --- | --- | --- | --- | --- | --- | --- | --- | --- | --- | --- | --- | --- | --- |
|  | Adjusted OR (95% CI) | | *p for Inter-action* |  | Adjusted OR (95% CI) | | *p for Inter-action* |  | Adjusted OR (95% CI) | | *p for Inter-action* |  | Adjusted OR (95% CI) | | p for Inter-action |
|  | < 45y | ≥ 45y |  |  | Male | Female |  |  | Yes | No |  |  | Yes | No |  |
|  | (n=798) | (n=596) |  |  | (n=735) | (n=659) |  |  | (n=254) | (n=1140) |  |  | (n=324) | (n=1070) |  |
| Metabolic disease |  |  | 0.381 |  |  |  | 0.491 |  |  |  | 0.453 |  |  |  | 0.971 |
| Quartile 1 | Ref | Ref |  |  | Ref | Ref |  |  | Ref | Ref |  |  | Ref | Ref |  |
| Quartile 2 | 1.25 (0.77-2.04) | 1.10 (0.57-2.13) |  |  | 1.47 (0.73-2.96) | 1.16 (0.72-1.89) |  |  | 2.56 (0.65-10.12) | 1.15 (0.76-1.75) |  |  | 1.81 (0.51-6.42) | 1.13 (0.74-1.72) |  |
| Quartile 3 | 0.88 (0.53-1.45) | 1.76 (0.85-3.65) |  |  | 1.50 (0.78-2.90) | 0.94 (0.54-1.61) |  |  | 3.09 (0.84-11.45) | 1.00 (0.64-1.54) |  |  | 1.36 (0.43-4.34) | 1.09 (0.70-1.70) |  |
| Quartile 4 | 3.12 (1.77-5.51) | 2.94 (1.23-7.04) |  |  | 4.37 (2.04-9.35) | 2.52 (1.33-4.77) |  |  | 6.07 (1.47-25.12) | 2.87 (1.72-4.80) |  |  | 4.07 (1.11-14.96) | 3.01 (1.78-5.08) |  |
| 1 vs 0 component |  |  | 0.288 |  |  |  | 0.871 |  |  |  | 0.581 |  |  |  | 0.906 |
| Quartile 1 | Ref | Ref |  |  | Ref | Ref |  |  | Ref | Ref |  |  | Ref | Ref |  |
| Quartile 2 | 1.28 (0.73-2.22) | 0.98 (0.43-2.25) |  |  | 1.44 (0.61-3.40) | 1.18 (0.68-2.04) |  |  | 1.37 (0.15-12.47) | 1.19 (0.74-1.92) |  |  | 1.34 (0.26-6.82) | 1.17 (0.72-1.90) |  |
| Quartile 3 | 0.77 (0.43-1.40) | 1.49 (0.60-3.68) |  |  | 1.21 (0.53-2.74) | 0.88 (0.46-1.69) |  |  | 3.14 (0.45-21.76) | 0.87 (0.52-1.45) |  |  | 1.19 (0.26-5.35) | 0.95 (0.56-1.61) |  |
| Quartile 4 | 2.42 (1.28-4.59) | 2.84 (1.02-7.92) |  |  | 3.32 (1.36-8.15) | 2.15 (1.05-4.39) |  |  | 7.07 (0.89-56.21) | 2.39 (1.34-4.26) |  |  | 2.91 (0.46-11.46) | 2.69 (1.49-4.84) |  |
| 2 vs 0 component |  |  | 0.815 |  |  |  | 0.648 |  |  |  | 0.376 |  |  |  | 0.642 |
| Quartile 1 | Ref | Ref |  |  | Ref | Ref |  |  | Ref | Ref |  |  | Ref | Ref |  |
| Quartile 2 | 1.27 (0.59-2.71) | 1.75 (0.76-4.04) |  |  | 1.80 (0.72-4.50) | 1.14 (0.54-2.44) |  |  | 6.25 (0.92-42.40) | 1.05 (0.57-1.93) |  |  | 3.34 (0.68-16.43) | 0.99 (0.53-1.83) |  |
| Quartile 3 | 0.82 (0.38-1.79) | 2.48 (0.99-6.22) |  |  | 1.83 (0.76-4.40) | 0.90 (0.37-2.16) |  |  | 9.02 (1.39-58.40) | 0.90 (0.47-1.71) |  |  | 2.73 (0.56-13.29) | 1.02 (0.53-1.95) |  |
| Quartile 4 | 2.61 (1.14-5.96) | 3.87 (1.34-11.18) |  |  | 4.12 (1.57-10.81) | 2.69 (1.04-6.91) |  |  | 13.71 (1.90-99.22) | 2.50 (1.24-5.05) |  |  | 5.44 (1.04-28.29) | 2.47 (1.20-5.09) |  |
| 3-6 vs 0 component |  |  | 0.278 |  |  |  | 0.716 |  |  |  | 0.604 |  |  |  | 0.464 |
| Quartile 1 | Ref | Ref |  |  | Ref | Ref |  |  | Ref | Ref |  |  | Ref | Ref |  |
| Quartile 2 | 1.82 (0.79-4.15) | 0.91 (0.41-2.05) |  |  | 1.29 (0.58-2.88) | 1.31 (0.59-2.94) |  |  | 2.41 (0.60-9.76) | 1.16 (0.61-2.19) |  |  | 1.81 (0.45-7.30) | 1.07 (0.56-2.04) |  |
| Quartile 3 | 2.02 (0.93-4.41) | 1.52 (0.65-3.57) |  |  | 1.67 (0.78-3.57) | 1.42 (0.61-3.35) |  |  | 2.82 (0.73-10.94) | 1.49 (0.80-2.80) |  |  | 1.33 (0.38-4.65) | 1.76 (0.94-3.30) |  |
| Quartile 4 | 6.67 (2.90-15.33) | 2.84 (1.08-7.53) |  |  | 5.12 (2.21-11.86) | 3.40 (1.26-9.19) |  |  | 7.14 (1.68-30.41) | 3.79 (1.88-7.65) |  |  | 5.47 (1.37-21.82) | 4.05 (1.98-8.28) |  |

Models were adjusted for age, gender, the number of RBCs, smoke, drink, exercise, education, and meat. OR, Odd Ratios; CI, confidence interval; Ref, reference.

Supplementary table 2. Subgroups analysis for the association between NAD^+^ levels and metabolic disease or its components.

|  | Age | | |  | Sex | | |  | Drink | | |  | Smoke | | |
| --- | --- | --- | --- | --- | --- | --- | --- | --- | --- | --- | --- | --- | --- | --- | --- |
|  | Adjusted OR (95% CI) | | *p for Inter-action* |  | Adjusted OR (95% CI) | | *p for Inter-action* |  | Adjusted OR (95% CI) | | *p for Inter-action* |  | Adjusted OR (95% CI) | | p for Inter-action |
|  | < 45y | ≥ 45y |  |  | Male | Female |  |  | Yes | No |  |  | Yes | No |  |
|  | (n=798) | (n=596) |  |  | (n=735) | (n=659) |  |  | (n=254) | (n=1140) |  |  | (n=324) | (n=1070) |  |
| Metabolic disease |  |  | 0.377 |  |  |  | 0.115 |  |  |  | 0.221 |  |  |  | 0.564 |
| <31.0 μmol/L | Ref | Ref |  |  | Ref | Ref |  |  | Ref | Ref |  |  | Ref | Ref |  |
| ≥31.0 μmol/L | 1.57 (1.08-2.29) | 1.48 (0.87-2.54) |  |  | 1.99 (1.19-3.33) | 1.28 (0.87-1.89) |  |  | 3.58 (1.35-9.50) | 1.41 (1.01-1.96) |  |  | 1.82 (0.72-4.62) | 1.47 (1.05-2.04) |  |
| 1 vs 0 component |  |  | 0.780 |  |  |  | 0.398 |  |  |  | 0.298 |  |  |  | 0.691 |
| <31.0 μmol/L | Ref | Ref |  |  | Ref | Ref |  |  | Ref | Ref |  |  | Ref | Ref |  |
| ≥31.0 μmol/L | 1.21 (0.79-1.85) | 1.47 (0.77-2.81) |  |  | 1.57 (0.85-2.89) | 1.12 (0.71-1.75) |  |  | 3.22 (0.77-13.47) | 1.18 (0.81-1.71) |  |  | 1.13 (0.37-3.52) | 1.28 (0.87-1.88) |  |
| 2 vs 0 component |  |  | 0.138 |  |  |  | 0.559 |  |  |  | 0.454 |  |  |  | 0.537 |
| <31.0 μmol/L | Ref | Ref |  |  | Ref | Ref |  |  | Ref | Ref |  |  | Ref | Ref |  |
| ≥31.0 μmol/L | 1.65 (0.92-2.96) | 1.47 (0.76-2.87) |  |  | 1.73 (0.90-3.31) | 1.47 (0.79-2.72) |  |  | 3.56 (0.98-12.98) | 1.34 (0.83-2.15) |  |  | 2.07 (0.66-6.51) | 1.34 (0.83-2.17) |  |
| 3-6 vs 0 component |  |  | 0.003 |  |  |  | 0.164 |  |  |  | 0.207 |  |  |  | 0.605 |
| <31.0 μmol/L | Ref | Ref |  |  | Ref | Ref |  |  | Ref | Ref |  |  | Ref | Ref |  |
| ≥31.0 μmol/L | 3.35 (1.82-6.18) | 1.37 (0.72-2.59) |  |  | 2.54 (1.39-4.63) | 1.66 (0.89-3.12) |  |  | 3.82 (1.35-10.77) | 1.90 (1.17-3.09) |  |  | 2.41 (0.86-6.77) | 2.05 (1.26-3.34) |  |

Models were adjusted for age, gender, the number of RBCs, smoke, drink, exercise, education, and meat. OR, Odd Ratios; CI, confidence interval; Ref, reference.
